# Novel Diagnostic Approaches for Assessment of the Clinically Negative Neck in Head and Neck Cancer Patients

**DOI:** 10.3389/fonc.2020.637513

**Published:** 2021-02-05

**Authors:** Daphne A. J. J. Driessen, Tim Dijkema, Willem L. J. Weijs, Robert P. Takes, Sjoert A. H. Pegge, Patrik Zámecnik, Adriana C. H. van Engen-van Grunsven, Tom W. J. Scheenen, Johannes H. A. M. Kaanders

**Affiliations:** ^1^ Department of Radiation Oncology, Radboud University Medical Center, Nijmegen, Netherlands; ^2^ Department of Oral- and Maxillofacial Surgery and Head and Neck Surgery, Radboud University Medical Center, Nijmegen, Netherlands; ^3^ Department of Otorhinolaryngology and Head and Neck Surgery, Radboud University Medical Center, Nijmegen, Netherlands; ^4^ Department of Medical Imaging, Radboud University Medical Center, Nijmegen, Netherlands; ^5^ Department of Pathology, Radboud University Medical Center, Nijmegen, Netherlands

**Keywords:** head and neck cancer, lymph node metastases, nodal staging, FDG-PET/CT, nanoparticles enhanced MRI, sentinel lymph node identification, elective neck treatment

## Abstract

In head and neck cancer, the presence of nodal disease is a strong determinant of prognosis and treatment. Despite the use of modern multimodality diagnostic imaging, the prevalence of occult nodal metastases is relatively high. This is why in clinically node negative head and neck cancer the lymphatics are treated “electively” to eradicate subclinical tumor deposits. As a consequence, many true node negative patients undergo surgery or irradiation of the neck and suffer from the associated and unnecessary early and long-term morbidity. Safely tailoring head and neck cancer treatment to individual patients requires a more accurate pre-treatment assessment of nodal status. In this review, we discuss the potential of several innovative diagnostic approaches to guide customized management of the clinically negative neck in head and neck cancer patients.

## Introduction

Head and neck squamous cell carcinoma (HNSCC) accounted for 890,000 new cases and 450,000 deaths worldwide in 2018 and overall 5-year survival is 50–60% (1, 2). At diagnosis, about one-third of all patients present with a clinically positive neck (3). Nodal involvement is associated with an increased risk of regional recurrences and distant metastases and thus an important prognostic factor and determinant for treatment (4, 5).

Treatment of the lymph node positive neck comprises neck dissection, (chemo)radiotherapy or a combination of these (6). The extent and method of treatment is determined by the location of the primary tumor, stage, and size of the metastatic nodes and by the patient’s age, performance status, and preference. It is general practice to treat the neck with the same modality as the primary tumor. Management of the clinically negative (cN0) neck, i.e., no identified nodal metastases after state-of-the-art diagnostic work-up, has been debated extensively. To determine the optimal treatment strategy for cN0 patients, a decision analysis model was introduced several decades ago. Elective neck treatment was considered indicated with a higher than 20% probability of subclinical nodal metastatic disease (7). This, by definition, results in overtreatment in 80% of cN0 patients (8, 9). Since then, newer decision models have been applied and conclusions vary (10, 11). Modern views on the management of the cN0 neck, however, focus less on cut-off values but value more individual, institutional and other relevant variables to optimize management of the neck (12).

Surgical neck treatments are associated with a serious morbidity profile including impaired shoulder function, post-operative pain and nerve damage (13). After radiotherapy to the neck there is dose-dependent risk of xerostomia, dysphagia, atherosclerosis of the carotids and hypothyroidism (14–16). Xerostomia and dysphagia are the most important negative predictors of quality of life and atherosclerosis of the carotids may cause ischemic brain infarctions and impair life expectancy (17–19). Therefore, de-intensifying therapy safely to avoid these sequelae should be a key focus of clinical research. To achieve this, more accurate pre-treatment assessment of neck status is required.

Recent advances in high resolution imaging modalities such as magnetic resonance imaging (MRI) and computed tomography (CT) as well as 18F-fluorodeoxyglucose positron emission tomography (FDG-PET) and ultrasound guided fine needle aspiration cytology (US-FNAC) have resulted in an improved accuracy in identifying nodal metastases. However, a major limitation of the radiological assessment of cervical lymph nodes is that it still relies on criteria such as size, signal intensity changes, shape, and rim, limiting its discriminative power (20, 21). Therefore, lymph nodes in the order of ≤5 mm containing small metastases are easily missed despite state-of-the-art imaging techniques.

The purpose of this review is to outline novel diagnostic applications for nodal assessment in HNSCC patients and to discuss their potential role in tailoring treatment of the clinically negative neck.

## FDG-PET/CT

The introduction of FDG-PET allowed functional evaluation of lymph nodes in addition to morphologic evaluation established by conventional imaging. Results of large meta-analyses conducted in 2013 and 2015 show superiority of FDG-PET/CT over conventional anatomical imaging for nodal staging (22, 23). Moreover, it was demonstrated that FDG-PET resulted in alteration of nodal treatment in approximately 1 out of 4 patients compared to conventional imaging; with nodal upstaging in 8–21% and downstaging in 3–11% (24–28).

With curative radiotherapy it is common practice to deliver a high dose to gross tumor locations and a lower “elective” dose to areas of presumed small tumor deposits. These include more distant areas of local tumor extensions, e.g., perineural or spidery growth and draining lymph node stations. This concept considers two manifestations of tumor, apparent gross tumor and small tumor deposits under the detection threshold of diagnostics. The “two dose level” principle has been employed successfully since the middle of the previous century (29, 30). Due to the technological advances in diagnostic imaging, smaller metastases are now better detected and are considered part of the “gross” tumor volume. Concurrently, the occult nodal metastatic load that needs elective treatment has decreased due to improvements in diagnostic accuracy of the neck with state-of-the-art imaging (31, 32). This indicates that nowadays unnecessary large areas with a decreasing tumor volume are being irradiated with a radiation dose that is likely to be higher than required, resulting in unintended dose-escalation or overtreatment. Since the dose required for tumor control is directly dependent on tumor load, dose prescription practice should be revised (33). The current “two dose level” concept can be replaced with a novel “gradient dose” concept in which dose is prescribed proportional to tumor load. Quantitative functional imaging with FDG-PET can help to guide such gradient-dose prescription because FDG-uptake can be a surrogate for metabolic activity of tumor cells and tumor load (34).

The randomized UPGRADE-RT trial (clinicaltrials.gov, NCT02442375) investigates this FDG-PET guided “gradient dose” concept and aims for a reduction of the elective radiation dose (30, 35). Based on FDG-uptake and two-dimensional size of lymph nodes, a risk assessment of harboring metastatic disease is made for every individual lymph node. Nodes that are considered negative receive a 20% lower than conventional elective dose (35 vs 45 Gy equivalent dose). Nodes that are borderline sized and have a moderate FDG-uptake will receive an “intermediate” dose of 60 Gy. Implications on safety, toxicity and quality of life are evaluated.

## USPIO-Enhanced MRI

### Mechanism of Action

**Figure 1:**
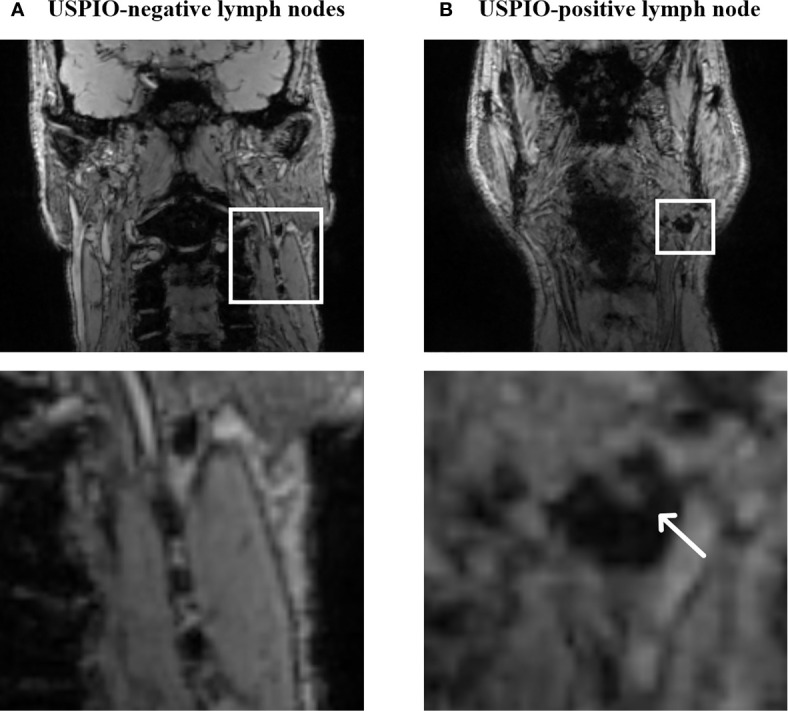
MR characteristics of USPIO-negative lymph nodes **(A)** and a partially USPIO-positive lymph node **(B)** on a T2*-weighted iron-sensitive sequence (own unpublished data). The white arrow in **(B)** points out an area of increased signal intensity suspicious for a metastasis.

The pioneering work of Weissleder and colleagues in 1990 led to the conception of ultrasmall particles of superparamagnetic iron oxide (diameter of 20–50 nm) (36). The small particle size and low molecular weight dextran surface coating lead to a characteristic biodistribution to the lymph nodes (37–39). USPIO delivery to the lymph nodes after intravenous infusion is established by two routes. First, USPIO passes through the high endothelial venules of lymph nodes to reach the nodal parenchyma. Here they are taken up by cells of the mononuclear phagocyte system (MPS) (36, 40). Second, the particles pass the endothelial layer of the capillaries into the tissue interstitium. From here they are transported to the lymph nodes *via* the lymphatic drainage system to be phagocytosed by the cells of the MPS. On MRI, healthy lymph nodes show a decrease in signal intensity on the multi gradient echo (mGRE) T2*-weighted sequence due to magnetic susceptibility and T2 shortening effects of iron oxide (21, 37, 41). A preclinical study demonstrated restrained USPIO uptake in metastatic lymph nodes due to a lesser presence of cells of the MPS and thus iron. Consequently, the MR signal is maintained on imaging (40). This was the first study to show a distinction between benign and malignant lymph nodes based on differences in MR signal intensity after USPIO infusion, which is visualized in an example from our own experience (**Figure 1**).

The coated iron compounds are manufactured as a lyophilized powder (Ferumoxtran-10, Ferrotran^®^ SPL Medical B.V. Nijmegen, the Netherlands). Dose prescription is according to body weight, 2.6 mg of iron per kilogram bodyweight is diluted in 100 ml of 0.9% saline solution for slow-drip intravenous administration over 30–45 min (42). Timing of postcontrast MR imaging of the cervical region has been evaluated and the optimal interval after infusion is established at 24 to 36 h (43).

Recent safety data from 310 prostate cancer patients who, between January 2014 and July 2016, underwent USPIO-enhanced MRI in a slow-drip fashion showed that adverse effects occurred in 8 out of 310 (2.6%) patients of which 7 (2.3%) were definitely or possibly USPIO-related (44). These were back pain, flushing, nausea, and a dry mouth and were all mild in nature (grade 1).

### Diagnostic Value of USPIO-Enhanced MRI

The first study to detect cervical lymph node metastases with USPIO-enhanced MRI in humans was performed in the mid-nineties. Encouraging sensitivity and specificity rates of 95 and 84% were reported (45). From the data provided in the publication, PPV and NPV of 83 and 84% can be derived. Several prospective studies were subsequently performed until 2009, yielding values for sensitivity, specificity, PPV and NPV ranging from 82–100%, 77–100%, 8–100%, and 90–100%, respectively (**Table 1**) (21, 38, 46–51). The poor lower bound of 8% for PPV originates from a study with a small sample size (n = 11) of T1-2 oral cavity squamous cell carcinoma patients in which only one lymph node in the whole study population was proven metastatic (51). Furthermore, only standard histopathologic examination was performed in this study without immunohistochemistry and multi-slice sectioning of the lymph nodes, possibly missing small metastatic lesions (51). In addition, some difficulties in the interpretation of USPIO-MR images of the head and neck region leading to false-positive results were reported (51). The level I lymph nodes draining the oral mucosa frequently exhibit inflammatory changes resulting from oral and dental infections and exposure to foreign material. In case of an inflammatory process, histopathological alterations in lymph nodes are described such as hyperplasia of germinal centers, hyaline metamorphosis, fibrosis and granulomatosis (37, 38, 49, 51, 52). These can modify USPIO-uptake due to changes in the migration and distribution of the cells of the MPS and can be a source of error in accurate detection of nodal metastases. Characteristics of USPIO-uptake in inflammatory lymph nodes need to be elucidated by studies using immunohistochemistry and cohorts including sufficient numbers of patients with ulcerating tumors or other sources of inflammation.

**Table 1:** Studies exploring the diagnostic accuracy of USPIO-enhanced MRI for nodal detection in HNSCC.

Author	Year	Number of nodes in analysis	Number of patients in analysis	Clinical N-stage	Dose (mg Fe/kg body weight)	MRI field strength in Tesla (no.)	MRI timing in hours	Sensitivity (%)	Specificity (%)	PPV (%)	NPV (%)
Anzai (45)	1994	91	11	n.p.	1.7	1.5	10–12 (4) 22–24 (8) 48 (2)^a^	95	84	n.p.	n.p.
Hoffman (46)	2000	101	9	8/9 cN+ 1/9 cN-	1.7	1.5	24–36	95	99	n.p.	n.p.
Mack (21)	2002	1029	27	29/30 cN+ 1/30 cN-	2.6	1.5	24–36	86	100	100	99
Sigal (38)	2002	86	81	53/81 cN+ 28/81 cN-	2.6	1.5 (78) 1.0 (3)	24–36	≥ 88	≥ 77	≥ 51	≥ 90
Anzai (47)	2003	n.p.	29	n.p.	2.6	n.p.	24–36	96	87	n.p.	n.p.
Baghi (48)	2005	363	26	17/28 cN+ 11/28 cN-	2.6	1.5	24–36	82.3	100	100	93
Curvo-Semedo (49)	2006	63	19	13/20 cN+ 7/20 cN-	2.6	1.5	24–36	96	78.9	75	96.8
Baghi (50)	2007	80	17	8/17 cN+ 9/17 cN-	2.6	n.p.	24–36	n.p.	94	n.p.	n.p.
Wensing (51)	2011	143	9	0/9 cN+ 9/9 cN-	2.6	1.5 3.0	24–36	100 (1.5T)	92 (1.5 T)	8 (1.5 T)	100 (1.5 T)
						3.0	24–36	100	93	9&10	100

a 2 patients underwent MRI twice at 10 and 24 h.

cN-, clinically node negative; cN+, clinically node positive; Fe, iron; kg, kilogram; MRI, magnetic resonance imaging; N, node; mg, milligram; n.p., not provided; n.s., not specified; no., number of patients; NPV, negative predictive value; PPV, positive predictive value.

Although the available data are promising, they are outdated and research was performed with older 1.5 Tesla (T) MR techniques. Optimization of the latest three-dimensional iron-sensitive sequences on 3T MR systems will provide a higher spatial resolution in imaging, potentially favoring diagnostic performance in smaller nodes when enhanced with USPIO. T2*-weighted GRE imaging of the head and neck area used to be vulnerable to susceptibility artefacts from air-tissue interfaces, potentially limiting its value in this particular area. However, due to recent technological developments that resulted in an increased spatial resolution and the use of both shorter and multiple echo times, this is no longer an issue. The diagnostic potential of USPIO-enhanced MRI in HNSCC patients therefore requires validation using larger cohorts and modern MR-imaging methods. A prospective validation study (USPIO-NECK study) is ongoing in which head and neck cancer patients scheduled for neck dissection undergo USPIO-enhanced 3T MRI. MRI findings are correlated to histopathology on a node-to-node level, aided by ex-vivo MRI of the dissection specimen. Dissected lymph nodes are examined by immunohistochemistry and cells of the MPS capturing USPIO are characterized and are spatially correlated with metastatic deposits. This study is currently open for enrollment (clinicaltrials.gov, NCT03817307). Another ongoing study investigates the feasibility of USPIO-enhanced MRI for visualization of tumor spread in lymph node positive HNSCC patients. Eligible subjects undergo MRI 24–72 h after intravenous USPIO infusion. The study is regarded feasible if good quality images (subjectively assessed) can be obtained in at least 75% of the participants. MRI results are not validated with histopathological results in this study (clinicaltrials.gov studyID: NCT01895829).

### Availability

Evidence regarding the potential diagnostic application of USPIO-enhanced MRI in various tumor subsites became available since the 1990s. In 2006, the manufacturer (AMAG Pharmaceuticals Inc., Waltham, MA, USA) and its European partner (Guerbet, Villepinte, France) attempted to register ferumoxtran-10 for marketing authorization. After their application, the regulatory agencies [Food and Drug Administration (FDA) and European Medicine Agency (EMA)] concluded that, despite a good safety profile, the data were considered insufficient to unequivocally demonstrate the efficacy of ferumoxtran-10. As a consequence, the manufacturer withdrew the application of ferumoxtran-10 in 2007. SPL Medical B.V. (Nijmegen, the Netherlands) acquired all rights and records concerning the production of ferumoxtran-10 in 2015 (44). Since then the agent is available again for both clinical and scientific purposes. In addition, an international multi-center phase 3 pivotal trial has been initiated to register USPIO-enhanced MRI for the detection of nodal involvement in prostate cancer which can lead to more widespread production and clinical use (clinicaltrials.gov, NCT04261777) (53).

## Sentinel Lymph Node Detection

An important contemporary technique for the detection of occult nodal disease in HNSCC is the sentinel lymph node procedure (SLNP). The fundamental principle of the SLNP is a minimally invasive diagnostic tool to assess the first draining lymph nodes of the primary tumor site. The procedure consists of a preoperative phase in which ^99m^Tc radioisotope, a radioactive tracer, is injected in close proximity of the primary tumor followed by SPECT-CT. The imaging in combination with per-operative hand-held gamma probe detection of the tracer is used to identify and remove the sentinel lymph nodes. Histopathological analysis including step serial sectioning and immunohistochemistry will confirm the presence or absence of (microscopic) metastatic disease. If nodal involvement is ascertained, this is generally considered as an indication for additional neck treatment. If the SLNs are free of tumor, neck treatment can be safely omitted (9, 54).

A meta-analysis addressing diagnostic accuracy of SLNP in early oral cancer was published in 2013 and estimated an overall sensitivity of 93% (55). SLNP was demonstrated to have the best performance compared to other diagnostic tools (CT, MRI, PET, US, and US-FNAC) when employed as a staging strategy in cN0 HNSCC (9). The Sentinel European Node Trial prospectively accrued a large cohort of 415 T1-2N0 oral squamous cell carcinoma patients undergoing SLNP without elective neck dissection for validation. A safety analysis was performed and revealed an overall survival, disease-free survival, and disease-specific survival of 88, 92, and 94%, respectively, after a follow-up period of three years. SLNP had a sensitivity of 86% and a NPV of 95% (56). SLNP is associated with decreased morbidity in terms of shoulder function in particular and has proven to be more cost-effective when compared to elective neck dissection (57–61). As SLNP is established to be beneficial and oncologically safe, it is offered routinely in many centers (56, 62).

However, there are also disadvantages to SLNP using radioactive tracers. Patients and physicians are exposed to radioactivity and the utility of ^99m^Tc radioisotope and SPECT is limited due to low spatial resolution (63). Poorer accuracy rates were demonstrated in patients with tumors in the floor of the mouth due to the “shine-through” effect of radioactivity and scatter originating from the primary injection site shading the SLN (64–67). Furthermore, this procedure requires surgical removal of the SLNs under general anesthesia for histopathologic examination.

### Sentinel Lymph Node Detection in Pharynx and Larynx Cancers

SLNP is increasingly under investigation for HNSCC at other subsites than the oral cavity. Fifty oropharyngeal, hypopharyngeal and laryngeal cT1-3N0 cancer patients received ^99m^Tc radioisotope injection *via* rigid endoscopy at the beginning of surgery. SLNs were identified during lymphadenectomy with a handheld gamma probe and dissected separately. A total of 42 patients had tumor free SLNs of which 41 patients had a pathologically negative neck. The remaining eight patients had tumor positive SLNs. Sensitivity of the procedure was 89% and NPV was 98% in this study (68). In a similar study, 13 consecutive patients with primary cT3-4aN0 laryngeal cancer underwent intraoperative ^99m^Tc radioisotope administration. SLNs were detected with a handheld gamma-probe in the neck dissection specimen shortly after surgical removal. Results showed a sensitivity and NPV of 80 and 87.5%, respectively (69). Together these studies support the feasibility of SLNP in laryngeal and pharyngeal carcinoma. Nevertheless, in both studies, imaging of radioactive tracer distribution was not possible since ^99m^Tc radioisotope injection and surgery were scheduled in a single session. Mapping of the lymph drainage patterns was therefore not possible.

Performing a SLNP for this purpose recently also attracted the interest of radiation oncologists for the purpose of radiotherapy target volume determination (70). However, larynx and pharynx cancers are difficult to access for radioactive tracer injection and thereby demand SLNP to be performed under general anesthesia in most centers, currently limiting its application in this patient group.

**Figure 2:**
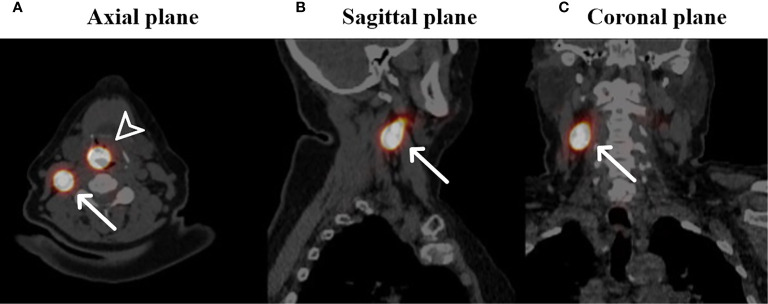
Fused SPECT-CT images of a patient with a cT1N0 squamous cell carcinoma located on the laryngeal side of the epiglottis who underwent SN identification (own unpublished data). Arrowhead: primary injection site of ^99m^Tc radioisotope, white arrows: identified SN in level II on the right side in the axial plane **(A)**, sagittal plane **(B)** and coronal plane **(C)**.

In the past few years, there has been significant progress with instrumentation *via* flexible endoscopy. Since the development of distal chip endoscopes with a working channel, diagnostic and therapeutic interventions in the outpatient clinic for laryngeal and pharyngeal pathology are increasing fast. There is now good expertise and experience with office-based endoscopic biopsy taking and even laser surgery of pharynx and larynx cancers (71–73). Laryngeal biopsy, vocal cord injection, and laser surgery have been widely investigated and demonstrate good patient tolerability and both diagnostic and therapeutic accuracy comparable with that achieved with operating room-based procedures. Overall, office-based procedures result in a shorter procedural duration, a more rapid diagnostic process, reduced costs, and reduced health risks largely due to avoiding sedation or general anesthesia (72, 73). These developments provide the opportunity for SLN detection in the less accessible pharynx and larynx tumors. Guided by the flexible endoscopy, tracer injection can be performed under local anesthesia. Its feasibility was demonstrated in 20 and 45 cN0 larynx and hypopharynx cancer patients in 2008 and 2011, respectively (74, 75). The FLEX-NODE study currently investigates the feasibility of flexible endoscopy-guided peritumoral injection of ^99m^Tc radioisotope in larynx and pharynx tumors for visualization of SLNs by SPECT (**Figure 2**). Accrual is ongoing (clinicaltrials.gov, NCT04068636).

### Alternative Tracers for Sentinel Lymph Node Detection

Superparamagnetic iron oxides (SPIO) particles, which are larger (59 nm) in diameter compared to the USPIO-particles (20–50 nm), have also been proposed as a tracer for sentinel node detection (76). Identification of SPIO-enhanced SLNs by using a magnetometer (SentiMag^®^) during surgery, analogous to the gamma probe used in the standard SLN procedure with the ^99m^Tc radioisotope, is an alternative to the current SLNP to avoid radiation exposure (77, 78).

Various studies evaluating SPIO for SLN identification have been conducted in breast cancer patients. Data extracted from five clinical trials including a total of 804 cN0 breast cancer patients who underwent the SLN procedure with both SPIO and the standard method utilizing the radioisotope were pooled in a meta-analysis. Results show that SPIO was non-inferior compared to the standard method in terms of both SLN detection and identification of patients with cN+ disease (79). Recent work in a cohort of 40 vulvar cancer patients show similar results (80). The data for HNSCC remain limited but the feasibility of this technique was confirmed in 11 oral cancer patients. In this study, patients underwent solely SPIO injection for SLN identification. A total of 45 SLNs detected intraoperatively with the magnetometer were removed and an additional elective neck dissection was performed. Histopathological analysis revealed a metastatic SLN in two patients. The remaining non-sentinel lymph nodes in these patients appeared negative for metastatic deposits (81).

SPIO nanoparticles have also been proposed as a contrast agent for MR lymphography for SLN identification. In a preliminary report, three patients with cancer of the oral tongue underwent a sentinel node procedure by using both MRI with peritumoral SPIO contrast injection and ^99m^Tc-radioisotope lymphoscintigraphy (82). The SLNs visualized on the ^99m^Tc-radioisotope lymphoscintigraphy correlated with the MRI findings. A larger body of experience has been obtained in breast cancer. Evaluation of 102 consecutive breast cancer patients who underwent SPIO-enhanced MRI for SLN detection showed its capability in accurately staging the axillary sentinel lymph nodes. Assessment of lymph node status on a nodal basis showed a sensitivity, specificity, NPV and PPV of 81.5, 90, 94.2, and 71%, respectively. All patients with metastases larger than 2 mm were successfully identified. However, 40% of the patients with micrometastases (≤ 2 mm) were missed presumably due to MR spatial resolution which is limited to 2 mm (83). However, the clinical relevance of micrometastases in SLNs regarding outcome and consequences for treatment is arguable (84). A subsequent study evaluating the pattern of SPIO uptake in 33 positive SLNs obtained from 30 breast cancer patients showed that in lymph nodes containing metastases of >2 mm, the area of high signal intensity on SPIO-enhanced MRI correlated with the size of metastases identified by pathology (85). These early results indicate that SPIO-enhanced MRI is capable of non-invasive SLN assessment.

In another study, 26 consecutive T1-T4N0 oral cavity cancer patients underwent peritumoral injection with gadobutrol, a gadolinium-based contrast agent, followed by dynamic MRI. First draining lymph nodes showing enhancement on dynamic MRI were identified as SLN and marked with blue dye under ultrasound-guidance. Subsequently, elective neck dissection was performed including the nodes marked as SLN. In all patients, a total of 44 SLNs were found on MRI. Histopathologic examination of the dissected specimens showed 11 lymph node positive patients of which 10 (90.9%) were correctly identified by the SLNP (86). The strengths of this method include a high spatial resolution and obviating the use of radioactive tracers. Consistent results come from Honda et al. using similar study setups in comparable cohorts of patients which were administered with peritumoral iopamidol (Iopamidol 370; Bayer Healthcare, Osaka, Japan) injections followed by CT and injection of blue dye (87) or indocyanine green (88) for SLN identification.

The results indicate that these methods are able to visualize the exact location of the SLNs, the lymph vessels draining the primary tumor and the surrounding anatomy and are useful in SLNs detection without using radioactive tracers. An important disadvantage of gadobutrol and iopamidol is that the lymph nodes that contain these contrast agents cannot be identified during surgery. An additional procedure, i.e., injection with dye is necessary which can introduce errors in the identification of SLNs.

Several other potential tracers and imaging modalities for SLN identification have been suggested. An alternative radioactive agent, ^99m^Tc tilmanocept, is characterized by a rapid injection site clearance, high retention within the SLN and lesser drainage to higher echelon lymph nodes and can therefore be particularly beneficial in flour of mouth tumors to overcome the shine-through effect (89, 90). A multicenter study investigating ^99m^Tc tilmanocept for SLNP in a cohort of HNSCC patients yielded an overall accuracy rate of 98.8% in correctly determining the pathological lymph node status of the neck. A similar high accuracy rate was obtained for the flour of mouth carcinoma subgroup, supporting the hypothesis of a diminished shine though effect and thus an improved detection rate (91).

A recent study in rats showed that injection of ^99m^Tc-radiolabeled gold nanoparticles functionalized with mannose and SPECT are capable of lymph drainage mapping. An advantage of these nanoparticles is that mannose binds to macrophage mannose receptors which are abundantly present in lymphoid tissue and thus actively targets lymph nodes, potentially improving specificity of the technique (92).

## Future Clinical Implications

De-intensification of treatment in order to decrease morbidity without compromising efficacy is increasingly becoming a topic of interest in oncology. Advanced, non-invasive nodal staging in HNSCC can impose clinically relevant changes in therapeutic strategies that reduce treatment sequelae. These considerations unabatedly apply to the treatment of the neck, because the radiation dose and extent of neck surgery or irradiation can have a significant impact on quality of life. In this review, several diagnostic modalities capable of contributing significantly to this issue, were outlined.

FDG-PET/CT has shown promise in the assessment of marginally enlarged lymph nodes and their treatment with radiotherapy. It is conceivable that these nodes do not need a high boost dose as for larger nodal metastases but that an intermediate dose may suffice. Furthermore, the improved sensitivity of nodal metastases imaging justifies a de-escalation of the elective radiation dose. In addition, a consequence may also be that neck node levels that need elective treatment can be selected on a more individualized basis. Both alterations in dose prescription and treated volume of the neck are expected to result in reduced morbidity and improved quality of life (30).

Although not yet incorporated into daily clinical practice, USPIO-enhanced MRI is a promising method for evaluation of lymph node metastases. It does not merely demonstrate if a lymph node carries a small metastasis, but it also can show the exact location and size of the metastasis within the lymph node (93). Previous research has demonstrated that metastatic deposits as small as 2 millimeter can be visualized with high-resolution USPIO-enhanced MRI (94). Guided by the USPIO-enhanced MR-images, radiation treatment plans may thus be safely customized to more selective neck regions (93).

Lymph drainage mapping by ^99m^Tc radioisotope injection and SPECT-CT is becoming increasingly valuable for radiotherapy treatment planning. In the SUSPECT trial (clinicaltrials.gov, NCT03968679), elective treatment of the contralateral neck in larynx and hypopharynx tumors and more advanced oropharyngeal tumors could be safely omitted when no contralateral drainage was visualized (70). However, since the procedure is chiefly performed under general anesthesia, it is time-consuming and accompanied with increased costs and morbidity. However, it was demonstrated that the procedure can also be done by flexible endoscopy under topical sedation (74). The dual advantage of non-invasive SLN identification and SPECT-guided ipsilateral elective neck irradiation is a promising development associated with a large potential clinical benefit (70).

A future step in the de-intensification of radiation treatment of HNSCC patients is to completely omit elective neck irradiation, analogous to the surgical strategy with ^99m^Tc-radioisotope SLNP in oral cancer patients. Various tracers and imaging modalities used for visualization of SLNs and lymphatic networks with their own set of advantages and disadvantages have been described. Strategies capable of nodal staging without surgical removal of the SLNs, such as SPIO-enhanced MRI, are of particular interest. Hence, SLN imaging negative for metastatic deposits could select patients with negative SLNs and provides clinicians with the opportunity to refrain from elective radiotherapy to the uni- or bilateral neck. For oral cancer patients, SLN imaging positive for metastatic deposits enables elective neck dissection in the same session when surgery of the primary tumor is performed and thus obviates the need for a second session. Obviously, these opportunities need to be explored in well-controlled clinical studies.

## Conclusion

At present there is no unequivocal strategy to address the cN0 neck in HNSCC patients, leading to significant overtreatment in a large proportion of patients. The diagnostic value of FDG-PET/CT, USPIO-enhanced MRI, and sentinel lymph node mapping with non-radioactive tracers for lymph node assessment in HNSCC patients is promising and potentially capable of improved non-invasive nodal staging. It may guide surgeons and radiation oncologists to safely target their treatment on an individual patient level, reducing acute toxicity and long-term morbidity. The clinical validation of these developments is ongoing. Subsequent prospective trials investigating the efficacy and safety of de-escalating treatment of the neck guided by these techniques should confirm its benefit for clinical practice.

## Author Contributions

DD performed the literature search and wrote the article. TD, JK, and TS contributed significantly to discussion of the content and revision of the manuscript. WW, RT, SP, PZ, and AvE-vG critically reviewed the manuscript. All authors agree to be accountable for the content of the work. All authors contributed to the article and approved the submitted version.

## Conflict of Interest

PZ declares to be a Scientific Advisor and owner of options of SPL Medical B.V., the manufacturer of USPIO.

The remaining authors declare that the research was conducted in the absence of any commercial or financial relationships that could be construed as a potential conflict of interest.
